# 3-[2-(4,4-Dimethyl-2,6-dioxocyclo­hexyl­idene)hydrazin­yl]benzonitrile

**DOI:** 10.1107/S1600536810013164

**Published:** 2010-04-24

**Authors:** Naki Çolak, Didem Aksakal, Ömer Andaç, Orhan Büyükgüngör

**Affiliations:** a Hitit Üniversitesi, Fen-Edebiyat Fakültesi, Kimya Bölümü, Çorum, Turkey; b Ondokuz Mayis Üniversitesi, Fen-Edebiyat Fakültesi, Kimya Bölümü, 55200 Atakum, Samsun, Turkey; c Ondokuz Mayis Üniversitesi, Fen-Edebiyat Fakültesi, Fizik Bölümü, 55200 Atakum, Samsun, Turkey

## Abstract

The title compound, C_15_H_15_N_3_O_2_, contains benzonitrile and 4,4-dimethyl-2,6-dioxocyclo­hexyl­idene groups connected *via* a hydrazinyl group. The structure is in the hydrazone tautomeric form in the solid state. The benzonitrile and hydrazinyl groups (3-hydrazinylbenzonitrile) are essentially coplanar with an r.m.s. deviation of 0.016 Å. Intra­molecular N—H⋯O hydrogen bonding helps to stabilize the mol­ecular structure, and weak inter­molecular C—H⋯O hydrogen bonding is present in the crystal structure.

## Related literature

The title compound is a tautomeric form of the azo compound; for the applications of azo compounds, see: Kobrakov *et al.* (2004 ▶); Karcı *et al.* (2004 ▶); Gale *et al.* (1998 ▶). For related structures of hydra­zone derivatives, see: Kelemen *et al.* (1982 ▶); Saylam *et al.* (2008 ▶); Seferoğlu *et al.* (2008 ▶; 2009 ▶); Batchelor *et al.* (1997 ▶); de Lima *et al.* (2009 ▶); de Souza *et al.* (2010 ▶); Özbey *et al.* (1997 ▶); Alpaslan *et al.* (2005 ▶). For additional structural analaysis, see: Spek (2003 ▶).
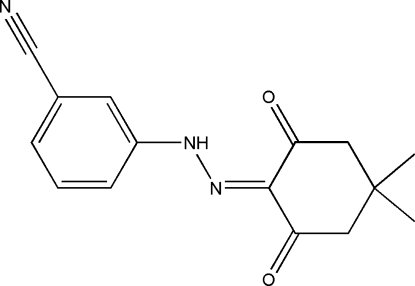


## Experimental

### 

#### Crystal data

C_15_H_15_N_3_O_2_
*M* *_r_* = 269.30
Orthorhombic, 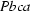

*a* = 12.9496 (8) Å
*b* = 8.6028 (6) Å
*c* = 24.324 (2) Å
*V* = 2709.8 (3) Å^3^
*Z* = 8
Mo *K*α radiation
μ = 0.09 mm^−1^
*T* = 296 K
0.80 × 0.36 × 0.14 mm

#### Data collection

Stoe IPDS II diffractometer
Absorption correction: integration (*X-RED32*; Stoe & Cie, 2002 ▶) *T*_min_ = 0.959, *T*_max_ = 0.991
10586 measured reflections
2880 independent reflections
1557 reflections with *I* > 2σ(*I*)
*R*_int_ = 0.052

#### Refinement

*R*[*F* ^2^ > 2σ(*F* ^2^)] = 0.044
*wR*(*F* ^2^) = 0.090
*S* = 0.85
2880 reflections
185 parameters
H atoms treated by a mixture of independent and constrained refinement
Δρ_max_ = 0.12 e Å^−3^
Δρ_min_ = −0.21 e Å^−3^

### 

Data collection: *X-AREA* (Stoe & Cie, 2002 ▶); cell refinement: *X-AREA*; data reduction: *X-RED32* (Stoe & Cie, 2002 ▶); program(s) used to solve structure: *SHELXS97* (Sheldrick, 2008 ▶); program(s) used to refine structure: *SHELXL97* (Sheldrick, 2008 ▶); molecular graphics: *ORTEP-3 for Windows* (Farrugia, 1997 ▶); software used to prepare material for publication: *SHELXL97*.

## Supplementary Material

Crystal structure: contains datablocks I, global. DOI: 10.1107/S1600536810013164/xu2749sup1.cif

Structure factors: contains datablocks I. DOI: 10.1107/S1600536810013164/xu2749Isup2.hkl

Additional supplementary materials:  crystallographic information; 3D view; checkCIF report

## Figures and Tables

**Table 1:** Hydrogen-bond geometry (Å, °)

*D*—H⋯*A*	*D*—H	H⋯*A*	*D*⋯*A*	*D*—H⋯*A*
N2—H2⋯O2	0.95 (2)	1.91 (2)	2.6461 (19)	132.6 (18)
C10—H10⋯O2^i^	0.93	2.57	3.442 (2)	156

Symmetry code: (i) 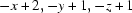.

**Table 2:** Selected bonds compared with related hydrazone compounds (Å)

N*sp*^3^—C*sp*^2^	N*sp*^2^—C*sp*^2^	N*sp*^3^—N*sp*^2^	Reference
1.412	1.313	1.305	Current work
1.406	1.313	1.300	Alpaslan *et al.* (2005 ▶)
1.382	1.289	1.364	de Lima *et al.* (2009 ▶)
1.347	1.282	1.378	de Souza *et al.* (2010 ▶)
1.376–1.384	1.300–1.325	1.319–1.325	Özbey *et al.* (1997 ▶)

**Fig. 1.**
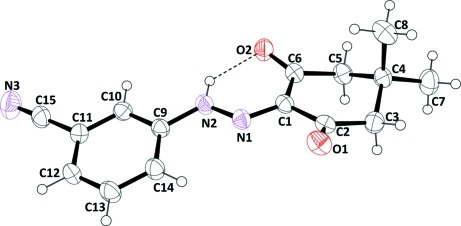
An ORTEPIII drawing of title complex with the atom numbering scheme at 40% ellipsoid. A view of the title compound, with the atom-labeling scheme.

**Fig. 2.**
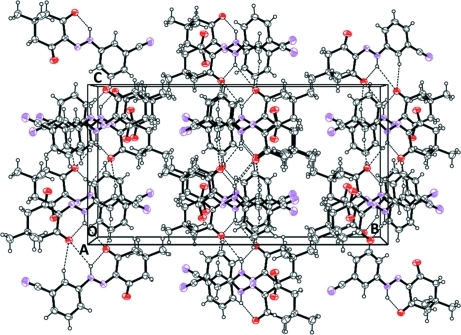
The packing diagram of the complex with hydrogen bonds shown as dashed lines.

**Fig. 3.**
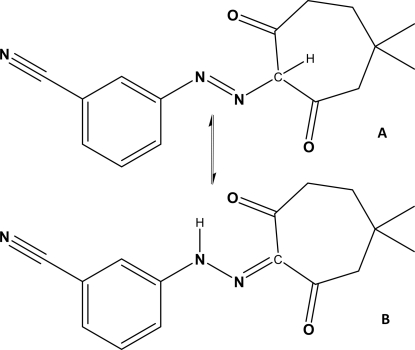
Tautomeric forms.

## supplementary crystallographic information

### Comment

It has been known for many years that the azo compounds are a widely used class of dyes due to their application in various fields such as the dyeing of textile fibers, the coloring of different materials, colored plastics and electrochemical sensors (Kobrakov *et al.*, 2004; Karcı *et al.*, 2004; Gale *et al.*, 1998).

Azo dyes are known to exist in the azo-hydrazone tautomeric forms (Saylam *et al.*, 2008; Seferoğlu *et al.*, 2008; Seferoğlu *et al.*, 2009). The dyes may exist in two possible tautomeric forms, namely azo form A and hydrazone form B as depicted in Figure 3. It is suggested that in a real azo compound the N=N double bond should have a length of 1.20–1.28 Å and the bond length of N–N single bonds, as in hydrazone tautomers, should be more than 1.4 Å (Kelemen *et al.*, 1982). In the title compound, N–N bond length is 1.304 Å, between the suggested N=N double bond and N–N single bond lengths. The bond lengths of N(sp3)–C(sp2), N(sp2)–C(sp2) and N(sp3)–N(sp2) in related hydrazone tatutomers are listed in Table 2. Comparing to related bond lengths in in Table 2, C1–N1(N(sp2)–C(sp2)) and N2–C9(N(sp3)–C(sp2)) are slightly longer, and N1–N2(N(sp3)–N(sp2)) is shorter than the expected values. Also, carbonyl oxygens slightly deviates from the least-squares plane (N1, C1, C2, C6) by -0.305 (3)Å for O1 and -0.297 (3)Å for O2. From the bond lengths and these deviations, it can be concluded that the compound exists both in azo and hydrazone tautomeric forms, and is mainly in the hydrazone tautomeric form, i.e. it is close to being real hydrazone pigments. C11–C15 bond length is longer for C(sp2)-C(sp1) but in agreement with previously reported value (Batchelor *et al.*, 1997).

### Experimental

A hydrochloric acid solution (2.5 ml) of 3-aminobenzonitrile (1.18 g, 0.010 mol) and an aqueous solution (10 ml) of sodium nitrite (0.69 g, 0.010 mol) were mixed and stirred at 273 K for 1 h, followed by the addition of ethanol solution (10 ml) of the coupling component 5,5-dimethylcyclohexane-1,3-dione (1.40 g, 0.010 mol) and continued stirring at 273 K for 4 h. The resulting product was filtered and washed with water, dried, and crystallized from ethanol gave fine crystals of benzonitrile, 3-[2-(4,4-dimethyl-2,6-dioxocyclohexylidene)hydrazinyl].

### Refinement

H atoms attached to carbon atoms were placed in calculated positions with U_iso_(H) = 1.2U_eq_(C). The coordinates of the amine hydrogen obtained from a difference map and refined isotropically.

### Crystal data

**Table:**

C_15_H_15_N_3_O_2_	*F*(000) = 1136
*M**_r_* = 269.30	*D*_x_ = 1.320 Mg m^−^^3^
Orthorhombic, *P**b**c**a*	Mo *K*α radiation, λ = 0.71073 Å
Hall symbol: -P 2ac 2ab	Cell parameters from 8472 reflections
*a* = 12.9496 (8) Å	θ = 1.6–27.3°
*b* = 8.6028 (6) Å	µ = 0.09 mm^−^^1^
*c* = 24.324 (2) Å	*T* = 296 K
*V* = 2709.8 (3) Å^3^	Prism, yellow
*Z* = 8	0.80 × 0.36 × 0.14 mm

### Data collection

**Table:**

Stoe IPDS II diffractometer	2880 independent reflections
Radiation source: fine-focus sealed tube	1557 reflections with *I* > 2σ(*I*)
plane graphite	*R*_int_ = 0.052
Detector resolution: 6.67 pixels mm^-1^	θ_max_ = 26.8°, θ_min_ = 1.7°
rotation method scans	*h* = −16→16
Absorption correction: integration (*X-RED32*; Stoe & Cie, 2002)	*k* = −10→7
*T*_min_ = 0.959, *T*_max_ = 0.991	*l* = −30→27
10586 measured reflections	

### Refinement

**Table:**

Refinement on *F*^2^	Primary atom site location: structure-invariant direct methods
Least-squares matrix: full	Secondary atom site location: difference Fourier map
*R*[*F*^2^ > 2σ(*F*^2^)] = 0.044	Hydrogen site location: mixed
*wR*(*F*^2^) = 0.090	H atoms treated by a mixture of independent and constrained refinement
*S* = 0.85	*w* = 1/[σ^2^(*F*_o_^2^) + (0.0393*P*)^2^] where *P* = (*F*_o_^2^ + 2*F*_c_^2^)/3
2880 reflections	(Δ/σ)_max_ < 0.001
185 parameters	Δρ_max_ = 0.12 e Å^−^^3^
0 restraints	Δρ_min_ = −0.21 e Å^−^^3^

### Special details

**Table:**

Geometry. All esds (except the esd in the dihedral angle between two l.s. planes) are estimated using the full covariance matrix. The cell esds are taken into account individually in the estimation of esds in distances, angles and torsion angles; correlations between esds in cell parameters are only used when they are defined by crystal symmetry. An approximate (isotropic) treatment of cell esds is used for estimating esds involving l.s. planes.
Refinement. Refinement of *F*^2^ against ALL reflections. The weighted *R*-factor wR and goodness of fit *S* are based on *F*^2^, conventional *R*-factors *R* are based on *F*, with *F* set to zero for negative *F*^2^. The threshold expression of *F*^2^ > σ(*F*^2^) is used only for calculating *R*-factors(gt) etc. and is not relevant to the choice of reflections for refinement. *R*-factors based on *F*^2^ are statistically about twice as large as those based on *F*, and *R*- factors based on ALL data will be even larger.

### Fractional atomic coordinates and isotropic or equivalent isotropic displacement parameters (Å^2^)

**Table:**

	*x*	*y*	*z*	*U*_iso_*/*U*_eq_	
C1	0.80305 (13)	0.3690 (2)	0.40962 (7)	0.0361 (5)	
C2	0.74316 (13)	0.2806 (2)	0.36821 (7)	0.0389 (5)	
C3	0.79117 (13)	0.2623 (3)	0.31243 (8)	0.0488 (6)	
H3A	0.7759	0.3541	0.2908	0.059*	
H3B	0.7595	0.1741	0.2942	0.059*	
C4	0.90833 (12)	0.2382 (3)	0.31345 (7)	0.0397 (5)	
C5	0.95623 (13)	0.3737 (3)	0.34490 (7)	0.0435 (5)	
H5A	1.0302	0.3573	0.3473	0.052*	
H5B	0.9449	0.4685	0.3242	0.052*	
C6	0.91435 (13)	0.3956 (2)	0.40184 (7)	0.0386 (5)	
C7	0.95030 (15)	0.2359 (3)	0.25490 (8)	0.0609 (7)	
H7A	1.0241	0.2257	0.2559	0.091*	
H7B	0.9321	0.3309	0.2366	0.091*	
H7C	0.9211	0.1495	0.2353	0.091*	
C8	0.93372 (16)	0.0846 (3)	0.34174 (9)	0.0588 (6)	
H8A	1.0073	0.0732	0.3444	0.088*	
H8B	0.9058	0.0001	0.3207	0.088*	
H8C	0.9041	0.0838	0.3779	0.088*	
C9	0.72958 (12)	0.5914 (2)	0.52616 (7)	0.0370 (5)	
C10	0.77582 (13)	0.6709 (2)	0.56870 (7)	0.0405 (5)	
H10	0.8472	0.6696	0.5727	0.049*	
C11	0.71482 (13)	0.7530 (2)	0.60557 (7)	0.0406 (5)	
C12	0.60825 (14)	0.7562 (3)	0.59960 (8)	0.0450 (5)	
H12	0.5675	0.8108	0.6245	0.054*	
C13	0.56389 (14)	0.6779 (3)	0.55664 (8)	0.0504 (6)	
H13	0.4926	0.6804	0.5523	0.060*	
C14	0.62310 (13)	0.5957 (3)	0.51986 (8)	0.0453 (5)	
H14	0.5919	0.5431	0.4909	0.054*	
C15	0.76084 (15)	0.8376 (3)	0.65054 (8)	0.0503 (6)	
N1	0.74838 (11)	0.42725 (19)	0.45015 (6)	0.0387 (4)	
N2	0.79244 (12)	0.5078 (2)	0.48910 (6)	0.0397 (4)	
N3	0.79474 (14)	0.9049 (3)	0.68665 (8)	0.0735 (7)	
O1	0.65786 (9)	0.22775 (17)	0.37853 (5)	0.0507 (4)	
O2	0.96910 (9)	0.43863 (18)	0.44021 (5)	0.0494 (4)	
H2	0.8663 (18)	0.515 (3)	0.4890 (8)	0.074 (7)*	

### Atomic displacement parameters (Å^2^)

**Table:**

	*U*^11^	*U*^22^	*U*^33^	*U*^12^	*U*^13^	*U*^23^
C1	0.0351 (9)	0.0363 (13)	0.0368 (10)	0.0015 (8)	0.0046 (8)	0.0001 (9)
C2	0.0371 (9)	0.0341 (12)	0.0455 (11)	0.0054 (9)	0.0010 (8)	0.0010 (9)
C3	0.0438 (10)	0.0592 (17)	0.0434 (12)	0.0017 (10)	−0.0014 (8)	−0.0087 (11)
C4	0.0385 (9)	0.0420 (14)	0.0387 (10)	0.0006 (9)	0.0065 (8)	−0.0021 (10)
C5	0.0389 (10)	0.0464 (14)	0.0451 (11)	−0.0030 (9)	0.0089 (8)	−0.0006 (10)
C6	0.0384 (10)	0.0360 (13)	0.0414 (10)	0.0003 (9)	0.0040 (8)	−0.0010 (10)
C7	0.0595 (12)	0.077 (2)	0.0459 (12)	−0.0031 (13)	0.0112 (10)	−0.0101 (13)
C8	0.0562 (12)	0.0484 (17)	0.0716 (14)	0.0059 (11)	0.0163 (11)	0.0012 (13)
C9	0.0356 (9)	0.0393 (13)	0.0363 (10)	0.0030 (9)	0.0056 (7)	0.0026 (9)
C10	0.0335 (10)	0.0483 (15)	0.0398 (11)	0.0022 (8)	0.0017 (8)	0.0014 (10)
C11	0.0428 (10)	0.0431 (14)	0.0359 (10)	0.0019 (9)	0.0022 (8)	−0.0008 (10)
C12	0.0433 (10)	0.0483 (14)	0.0435 (11)	0.0081 (10)	0.0080 (8)	−0.0057 (11)
C13	0.0336 (9)	0.0605 (16)	0.0570 (13)	0.0036 (9)	0.0051 (9)	−0.0092 (12)
C14	0.0361 (10)	0.0544 (16)	0.0455 (11)	−0.0017 (10)	0.0026 (8)	−0.0101 (11)
C15	0.0438 (11)	0.0629 (17)	0.0443 (12)	0.0019 (10)	0.0076 (10)	−0.0035 (12)
N1	0.0400 (7)	0.0382 (11)	0.0377 (8)	0.0007 (8)	0.0046 (7)	−0.0002 (8)
N2	0.0347 (8)	0.0449 (12)	0.0394 (9)	0.0023 (7)	0.0049 (7)	−0.0047 (8)
N3	0.0686 (12)	0.097 (2)	0.0553 (12)	−0.0122 (12)	0.0018 (10)	−0.0218 (13)
O1	0.0372 (7)	0.0493 (10)	0.0656 (9)	−0.0065 (6)	0.0061 (6)	−0.0052 (8)
O2	0.0378 (7)	0.0661 (12)	0.0443 (8)	−0.0018 (7)	0.0005 (6)	−0.0118 (8)

### Geometric parameters (Å, °)

**Table:**

C1—N1	1.313 (2)	C8—H8A	0.9600
C1—C6	1.472 (2)	C8—H8B	0.9600
C1—C2	1.481 (3)	C8—H8C	0.9600
C2—O1	1.221 (2)	C9—C10	1.377 (2)
C2—C3	1.501 (2)	C9—C14	1.388 (2)
C3—C4	1.532 (2)	C9—N2	1.412 (2)
C3—H3A	0.9700	C10—C11	1.388 (2)
C3—H3B	0.9700	C10—H10	0.9300
C4—C7	1.524 (2)	C11—C12	1.388 (2)
C4—C8	1.526 (3)	C11—C15	1.442 (3)
C4—C5	1.526 (3)	C12—C13	1.370 (3)
C5—C6	1.499 (2)	C12—H12	0.9300
C5—H5A	0.9700	C13—C14	1.374 (3)
C5—H5B	0.9700	C13—H13	0.9300
C6—O2	1.229 (2)	C14—H14	0.9300
C7—H7A	0.9600	C15—N3	1.140 (2)
C7—H7B	0.9600	N1—N2	1.305 (2)
C7—H7C	0.9600	N2—H2	0.96 (2)
			
N1—C1—C6	124.40 (17)	H7A—C7—H7C	109.5
N1—C1—C2	115.10 (15)	H7B—C7—H7C	109.5
C6—C1—C2	120.35 (16)	C4—C8—H8A	109.5
O1—C2—C1	121.69 (17)	C4—C8—H8B	109.5
O1—C2—C3	121.42 (17)	H8A—C8—H8B	109.5
C1—C2—C3	116.87 (16)	C4—C8—H8C	109.5
C2—C3—C4	114.21 (15)	H8A—C8—H8C	109.5
C2—C3—H3A	108.7	H8B—C8—H8C	109.5
C4—C3—H3A	108.7	C10—C9—C14	120.13 (17)
C2—C3—H3B	108.7	C10—C9—N2	118.83 (15)
C4—C3—H3B	108.7	C14—C9—N2	121.04 (18)
H3A—C3—H3B	107.6	C9—C10—C11	119.38 (16)
C7—C4—C8	109.47 (18)	C9—C10—H10	120.3
C7—C4—C5	109.47 (16)	C11—C10—H10	120.3
C8—C4—C5	110.37 (17)	C12—C11—C10	120.55 (18)
C7—C4—C3	109.86 (16)	C12—C11—C15	118.70 (17)
C8—C4—C3	109.75 (16)	C10—C11—C15	120.75 (16)
C5—C4—C3	107.90 (16)	C13—C12—C11	119.15 (18)
C6—C5—C4	114.32 (16)	C13—C12—H12	120.4
C6—C5—H5A	108.7	C11—C12—H12	120.4
C4—C5—H5A	108.7	C12—C13—C14	121.05 (18)
C6—C5—H5B	108.7	C12—C13—H13	119.5
C4—C5—H5B	108.7	C14—C13—H13	119.5
H5A—C5—H5B	107.6	C13—C14—C9	119.74 (19)
O2—C6—C1	120.94 (16)	C13—C14—H14	120.1
O2—C6—C5	122.05 (15)	C9—C14—H14	120.1
C1—C6—C5	116.97 (16)	N3—C15—C11	178.2 (2)
C4—C7—H7A	109.5	N2—N1—C1	120.80 (15)
C4—C7—H7B	109.5	N1—N2—C9	118.82 (16)
H7A—C7—H7B	109.5	N1—N2—H2	118.0 (13)
C4—C7—H7C	109.5	C9—N2—H2	122.9 (13)
			
N1—C1—C2—O1	−20.3 (3)	C4—C5—C6—C1	−38.1 (2)
C6—C1—C2—O1	163.84 (18)	C14—C9—C10—C11	1.0 (3)
N1—C1—C2—C3	158.29 (18)	N2—C9—C10—C11	−179.69 (19)
C6—C1—C2—C3	−17.5 (3)	C9—C10—C11—C12	−0.4 (3)
O1—C2—C3—C4	−143.91 (19)	C9—C10—C11—C15	−179.95 (19)
C1—C2—C3—C4	37.5 (3)	C10—C11—C12—C13	−0.3 (3)
C2—C3—C4—C7	−174.81 (19)	C15—C11—C12—C13	179.2 (2)
C2—C3—C4—C8	64.8 (2)	C11—C12—C13—C14	0.5 (3)
C2—C3—C4—C5	−55.5 (2)	C12—C13—C14—C9	0.0 (3)
C7—C4—C5—C6	175.37 (17)	C10—C9—C14—C13	−0.8 (3)
C8—C4—C5—C6	−64.1 (2)	N2—C9—C14—C13	179.9 (2)
C3—C4—C5—C6	55.8 (2)	C6—C1—N1—N2	−4.2 (3)
N1—C1—C6—O2	20.1 (3)	C2—C1—N1—N2	−179.81 (17)
C2—C1—C6—O2	−164.49 (19)	C1—N1—N2—C9	168.70 (18)
N1—C1—C6—C5	−157.67 (19)	C10—C9—N2—N1	177.20 (18)
C2—C1—C6—C5	17.8 (3)	C14—C9—N2—N1	−3.5 (3)
C4—C5—C6—O2	144.2 (2)		

### Hydrogen-bond geometry (Å, °)

**Table:**

*D*—H···*A*	*D*—H	H···*A*	*D*···*A*	*D*—H···*A*
N2—H2···O2	0.95 (2)	1.91 (2)	2.6461 (19)	132.6 (18)
C10—H10···O2^i^	0.93	2.57	3.442 (2)	156

Symmetry codes: (i) −*x*+2, −*y*+1, −*z*+1.

### Table 2 Selected bonds compared with related hydrazone compounds (Å) [should this be 1.313 and 1.305 for current work?]

**Table:**

Nsp^3^—Csp^2^	Nsp^2^—Csp^2^	Nsp^3^—Nsp^2^	Reference
1.412	1.313	1.305	Current work
1.406	1.313	1.300	Alpaslan *et al.* (2005)
1.382	1.289	1.364	de Lima *et al.* (2009)
1.347	1.282	1.378	de Souza *et al.* (2010)
1.376-1.384	1.300-1.325	1.319-1.325	Özbey *et al.* (1997)
